# Restoration after the Hand Is Completely Separated from the Arm

**Published:** 1880-09-20

**Authors:** L. L. Staton

**Affiliations:** N. C.


### RESTORATION AFTER THE HAND IS COMPLETELY SEPARATED FROM THE ARM.

BY L. L. STATON, M.D., N. C.

I desire to place on record the following, unusual, and so far as I know, the only case of the kind ever given to the profession:

On Friday afternoon, February 5th, 1880, I was called to see Mary Sumlin, a white girl aged eleven years, quite anæmic and rather small for one of her age. While helping her mother to procure fire-wood she placed her hand in the way of an axe, and at one blow had it severed from the styloid process diagonally across the trapezium passing through the scaphoid bone and posterior annular ligament, dividing all the muscles, bones and blood-vessels, and completely separating the hand from the arm excepting a small portion of skin, below the articulation, with the ulna, the hand was hanging at right angles to the arm when I saw her, about thirty minutes after the accident.

I determined at once upon amputation, at the joint above (the wrist), so returned to my office, a distance of a half mile, to procure the assistance of another physician; but finding this impracticable, I proceeded carefully to replace the hand, which was held securely in position with silver wire sutures and adhesive plaster.

In dressing the wound the patient complained of pain when I used the needle in the arm, but none when it was used in the hand.

I secured the hand and arm upon a broad splint and directed that they be kept warm by being wrapped in hot flannel cloths.

I saw her twelve hours afterwards; the hand was very much swollen; no sensation or pulsation could be detected nor had she complained of any pain, but rested quietly during the night.

Saw her the next day. She now complained of a little pain, but the hand and arm presented the same appearance as of yesterday.

Saw her upon the third day. Could now plainly feel pulsation in the hand; it had changed its color, and I now for the first time thought it possible to save the hand. From this time she did not have a bad symptom, nor was there any suppuration or secretions of any kind, the wound healed entirely by first intention.

I removed the sutures upon the fourteenth day, and afterwards she carried the hand in a sling and is now able to extend the fingers and grasp with nearly the usual strength. . There is no anchylosis of the wrist joint as I expected.—*N. C. Med. Journal.*

